# Westminster Hospital

**Published:** 1889-10-12

**Authors:** 


### WESTMINSTER HOSPITAL.

Dr. Phineas Abraham gave the address. The lecturer commenced by saying a few words in favour of the hospital. In a small school there was no danger of the student being lost in a crowd. The proportion of students passing their examinations from this hospital compares most favourably with the number from any other medical school. But examinations are not everything, for the medical man has always to be learning, observing, and judging. Dr. Abraham considers that those who aspire to become members of the profession should show evidence of a good general education, and the medical course should extend for at least five years. The fact that quacks had to hold their heads low, spoke well for the growing enlightenment of public opinion. With this, however, we cannot quite agree, for it appears to us that quackery is still rampant in the land. The lecturer desired to enter a plea for a more thorough study of physiology on the part of medical students. Lastly, Dr. Abraham offered some practical suggestions in reference to lectures, note-taking, etc., for "it is a mistake to depend on books rather than on practical work."

